# Correction: The effect of the gelation temperature on the structural, magnetic and magnetocaloric properties of perovskite nanoparticles manufactured using the sol–gel method

**DOI:** 10.1039/d4ra90071e

**Published:** 2024-06-21

**Authors:** Line Karoui, Mourad Smari, Taoufik Mnasri, Anna Bajorek

**Affiliations:** a Laboratory of Technology, Energy and Innovative Materials, TEMI, Department of Physics, Faculty of Sciences of Gafsa, University of Gafsa Tunisia linekaroui5@gmail.com; b Applied Physics Laboratory, Faculty of Sciences of Sfax, University of Sfax B.P. 1171 3000 Sfax Tunisia; c A. Chełkowski Institute of Physics, University of Silesia in Katowice 75 Pułku Piechoty 1 Chorzów 41-500 Poland

## Abstract

Correction for ‘The effect of the gelation temperature on the structural, magnetic and magnetocaloric properties of perovskite nanoparticles manufactured using the sol–gel method’ by Line Karoui *et al.*, *RSC Adv.*, 2024, **14**, 11456–11469, https://doi.org/10.1039/D4RA01086H.

The authors regret the omission of one of the authors, Anna Bajorek, from the original manuscript. The corrected list of authors and affiliations for this paper is as shown herein.

In addition, the Acknowledgements section should be corrected as follows.

The authors would like to acknowledge the funding of the magnetic measurements from the statutory subsidies for research activities of the Condensed Phase Research Team in the A. Chełkowski Institute of Physics, University of Silesia in Katowice, Poland.

The Royal Society of Chemistry apologises for these errors and any consequent inconvenience to authors and readers.

